# Effect of match location on the playing style of teams coached by ‘Pep’ Guardiola

**DOI:** 10.3389/fpsyg.2024.1502199

**Published:** 2024-11-13

**Authors:** Luis Pueyo, Víctor Murillo, Javier Álvarez, Alberto Sarmiento, Mario Amatria

**Affiliations:** ^1^Department of Physiatry and Nursing, University of Zaragoza, Zaragoza, Spain; ^2^Faculty of Educational Science, Pontifical University of Salamanca, Salamanca, Spain

**Keywords:** football, performance analysis, observational methodology, polar coordinates, team sport, playing style

## Abstract

**Introduction:**

Analysis in football seeks to find the performance factors that bring teams closer to success.

**Methods:**

This study aims to analyze the playing styles of two teams managed by Pep Guardiola (F.C. Barcelona and Manchester City) based on match location (home or away). Two methods of analysis were used: descriptive statistics through chi-square tests to evaluate game characteristics and the polar coordinates technique to analyze the relationships between the different lines of each team (goalkeeper, defenders, midfielders, and forwards).

**Results:**

The results showed that F.C. Barcelona maintained a consistent playing style regardless of location, exhibiting significant differences only in actions that involved shots or header (*p* = 0.035), with better performance at home. In contrast, Manchester City displayed significantly different performance in action success (*p* < 0.001), level of play elaboration (*p* = 0.004), density (*p* = 0.033), duration (*p* = 0.036), and actions that included a shot (*p* = 0.001) depending on the location. Additionally, qualitative analyses revealed differences in the relationships among the team lines according to match location, with Manchester City displaying more variability in these interactions than F.C. Barcelona.

**Discussion:**

The study concludes that although Guardiola applies a consistent set of strategies, match location has a greater influence on Manchester City’s performance, suggesting that this team adjusts its playing style on the basis of contextual conditions. These findings highlight the importance of considering factors such as location when preparing tactics to increase the probability of success in elite football.

## 1 Introduction

Football, in its evolution as a sport and cultural phenomenon, underwent significant changes in playing style throughout the 20th and early 21st centuries. Initially, focused on defensive tactics and counterattacks, modern strategies have evolved toward a more balanced approach that values ball possession, progressive buildup from the defense, and complex offensive plays (Barreira et al., 2015). The transformation of football dynamics is due not only to advancements in player technique and performance but also to adaptations to contextual variables such as match location, tournament category, match timing, and the level of the opposing team (Diana et al., 2017; Fernández-Navarro et al., 2018; González-Rodenas et al., 2020). Consequently, today’s elite football players must develop high versatility and motor competence, as well as the ability to process information and make decisions quickly and effectively to successfully overcome these challenges (Wallace and Norton, 2014).

Observational methodology (Anguera, 1979) has proven effective for conducting studies of sports behaviors that occur in their natural contexts (Anguera et al., 2017). Thanks to its non-intrusive nature and respect for behavioral spontaneity, it allows for the evaluation and analysis of interaction relationships among different players, as well as the behaviors that emerge from them at both individual and collective levels. Through this methodology, it is possible to identify various analytical techniques applicable in the sports world (Anguera and Hernández-Mendo, 2015), which have led to significant findings. These techniques have already been applied in previous football studies (Castañer et al., 2013; Pic, 2018) with meaningful applied results, enabling important contributions regarding team performance indicators (Mićović et al., 2023) and the playing styles developed according to different contexts (Castellano and Pic, 2019), thus aiding in the search for strategies that lead to success in elite football.

One of the most studied variables in this regard has been match location (Brito de Souza et al., 2019; Kong et al., 2022) and how it affects team performance. Playing a home match impacts various factors that involve different ways of playing. For example, Almeida et al. (2014) reported that home teams, compared with away teams, tend to defend in more advanced areas of the field, and this approach is more effective when analyzing higher-ranked teams versus those whose standing is lower. Similarly, Diana et al. (2017) identified more complex and elaborate attacking patterns in home matches than in away games. Lago-Peñas and Lago-Ballesteros (2011) even showed that home teams exhibit superior performance in terms of technical and tactical execution. In general, playing at home provides an advantage, and teams may severely alter their playing style on the basis of location (Sarmento et al., 2014).

La Liga and the Premier League offer paradigmatic examples of contexts that, despite sharing certain similarities, present distinctive characteristics and playing styles (Cooper and Pulling, 2020; Nagy et al., 2023). While La Liga has traditionally been known for its technical, possession-based football, the Premier League is noted for its fast pace and emphasis on physicality. Comparisons between these two competitions have been conducted on numerous occasions (Fernández-Navarro et al., 2016; González-Rodenas et al., 2021; Gouveia et al., 2023).

Within this framework, the figure of Pep Guardiola stands out as a unique case study. Having coached teams in both leagues and achieved success in each provides an ideal context to explore how a consistent set of strategies and tactical philosophies can manifest and adapt in leagues with such different characteristics. Previous studies (Immler et al., 2021) have compared playing styles among elite coaches, but few works have examined the evolution or transformation of a coach’s team playing characteristics over time across different competitions, with match location as the object of study.

The purpose of the present research was to evaluate the playing styles of two teams coached by Pep Guardiola (F.C. Barcelona and Manchester City) based on match location (home or away). Descriptive statistics, through chi-square tests, were used to understand their playing characteristics, and qualitative analysis, using the technique of polar coordinates, was applied to discover the interline relationships they produced (goalkeeper -GK-, defenders -DEF-, midfielders -MID-, and forwards -FW-) within each team’s formations. Thus, the aim was to determine whether these teams maintained a stable playing style regardless of where they played their matches or if they were compelled to adapt their match tactics.

## 2 Materials and methods

The present study corresponded to an observational design of the nomothetic, punctual, and multidimensional type (N/P/M): nomothetic because the two teams were observed as different observational units; punctual because a count of the players’ actions with the ball was made without aiming to conduct a global follow-up of the teams; and multidimensional due to the sequential heterogeneity of possibilities in the different game situations (Anguera et al., 2011).

### 2.1 Participants

An observational sampling with an intentional or convenience character was carried out (Otzen and Manterola, 2017) involving the two teams under study: F.C. Barcelona and Manchester City. The seasons in which both teams scored the highest number of league goals under Pep Guardiola’s management. At the start of the study these were the 2011/2012 season for F.C. Barcelona (114 goals) and the 2018/2019 season for Manchester City (95 goals). To enhance the validity of the sample, matches played against the top six teams at the end of the season in their national regular competitions were selected (Castellano et al., 2013), including both home and away games (Table 1).

**Table 1:** Observed matches of each team.

Matches of F.C. Barcelona 2011/2012	Matches of Manchester City 2018/2019
Valencia – Barcelona (A)	Arsenal – Manchester City (A)
Barcelona – Atlético de Madrid (H)	Liverpool – Manchester City (A)
Barcelona – Levante (H)	Tottenham – Manchester City (A)
Real Madrid – Barcelona (A)	Manchester City – Manchester United (H)
Málaga – Barcelona (A)	Chelsea – Manchester City (A)
Barcelona – Valencia (H)	Manchester City – Liverpool (H)
Atlético de Madrid – Barcelona (A)	Manchester City – Arsenal (H)
Levante – Barcelona (A)	Manchester City – Chelsea (H)
Barcelona – Real Madrid (H)	Manchester City – Tottenham (H)
Barcelona – Málaga (H)	Manchester United – Manchester City (A)

(H) = Home; (H) = Away (A).

The preparation of this manuscript did not require informed consent or the approval of any ethics committee, complying with the requirements of the National Commission for the Protection of Human Subjects of Biomedical and Behavioral Research (1979), as it involves observation of public footage where the subjects have no reasonable expectation of privacy and do not involve any staged intervention by the researcher or direct interaction with individuals. Similarly, notably, the fundamental ethical principles for research with human beings have been followed in accordance with the Declaration of Helsinki (World Medical Association, 2021; Bošnjak, 2001; Tyebkhan, 2003).

### 2.2 Observational instrument

The observation instrument used in this study is the one designed by Maneiro and Amatria (2018), developed as an ‘*ad hoc*’ instrument at its creation. It was based on a field format combined with category systems (Anguera et al., 2020) because the design is multidimensional and each of the criteria unfolds a list of categories, fulfilling the requirements of exhaustiveness and mutual exclusivity (Anguera and Hernández-Mendo, 2013).

Subsequently, once all ball-in-play actions were recorded, variables related to the developed playing style were examined, focusing on the success of the technical actions performed, the level of elaboration and density of the plays, duration, and the volume of shots or attempts (Table 2; Pueyo et al., 2024).

**Table 2:** Variables evaluated.

Variable	Definition	Measurement
Success of actions (González-Rodenas et al., 2023)	Qualitative evaluation of the result of each player’s technical interaction with the ball during game actions, considering an action successful if it subsequently reaches a teammate or ends in a goal. Actions that end in loss, game stoppage, or occasional interception are considered unsuccessful, in accordance with the criteria of the observational instrument.	Yes/No
Elaboration of offensive sequences (Amatria et al., 2019; Tenga et al., 2009)	Quantification of the total number of passes made in each offensive sequence, as an indicator of the level of construction and coordination of play.	0–1 (nonexistent); 2–3 (very low); 4–5 (low); 6–10 (medium); 11–15 (high); 16–20 (very high); 21 or more (maximum)
Density of offensive sequences (Amatria et al., 2019)	Measurement of the number of participants involved in each offensive sequence, providing a metric for the density of on-field collaboration.	0–1 (nonexistent); 2–3 (very low); 4–5 (low); 6–10 (medium); 11–15 (high); 16 or more (very high)
Duration of offensive sequences (Tenga and Sigmundstad, 2011)	Recording of the temporal duration, in seconds, of the offensive sequences to analyze their sustaining period.	0–5 s (low); 6–11 s (medium); 12 s or more (high)
Shot or header (Hewitt et al., 2016)	Recording and quantification of the occurrence of shots or attempts during offensive sequences, as an indicator of offensive completion.	Yes/No

### 2.3 Recording and coding

Data recording (Hernández-Mendo et al., 2014) was performed via the software Lince version 1.4 (Gabin et al., 2012). This software was used for recording and collecting all data, and multievents occurred, understanding the latter as each unit of record within the program. The data obtained are time-based and concurrent, that is, type IV (Bakeman, 1978). Subsequently, the GSEQ software version 5.1 (Bakeman and Quera, 2011) was used to perform sequential lag analysis. The data were introduced into a second program called Hoisan version 1.2 (Hernández-Mendo et al., 2012), with which the corresponding results of the polar coordinate analysis were obtained.

### 2.4 Data quality control

The researchers who participated in the observations of this study were both graduates in physical activity and sports sciences, with extensive experience as coaches in the context of football and in the development and application of observational methodology in this sport. Evidence of this is the previously published works by the manuscript’s authors (Álvarez Medina et al., 2020; Amatria et al., 2021; Pueyo et al., 2024).

To increase the quality of the data for this work, the principal investigator received specific training in the methodology and handling of the recording instrument, aligning with the guidelines proposed by Losada and Manolov (2015) as well as Anguera (2003). This emphasizes the importance of adequate training for the observer in studies of this nature, ensuring an appropriate level of competence and understanding in the research context.

To guarantee the validity of the data obtained through the observational instrument, the GSEQ software version 5.1 (Bakeman and Quera, 2011) was used. Through this software, Cohen’s Kappa coefficient (Cohen, 1960) was calculated a statistical measure that allowed for an intra-observer analysis in which the data records made at two distant time periods (1 week apart) were compared. Block 1 of the data evaluated corresponds to all the actions that make up the sample, whereas Block 2 comprises 15% of the recorded sequences (Arroyo et al., 2023). The concordance between these two blocks yielded a coefficient of 0.95 in the overall computation of the evaluated dimensions. Likewise, to provide greater robustness and achieve a higher degree of reliability, consultative concordance was carried out (Arana et al., 2016). This qualitative method eliminates the confusion generated by two different interpretations and consists of presenting to a second observer only the discrepant observations between both blocks of records without knowing to which one the recording error corresponds, generating a new data block (Block 3). The observer decides on the basis of their judgment, which of the records constitutes the definitive observation, thus overcoming any limitations inherent to intra-observer concordance. The levels of reliability obtained are within the range classified as “almost perfect” according to the criteria established by Landis and Koch (1977), thereby reinforcing the solidity of the results obtained in the research.

### 2.5 Data analysis

Two types of analyses were carried out in this study. On the one hand, a quantitative analysis was performed via Pearson’s chi-square statistic (χ^2^), following the formula: $\chi^{2}=\Sigma_{i=1\kappa }\;\Sigma_{j=1\kappa }\;\left[{\left(F_{ij}-F_{ij}\right)}^{2}/F_{ij}\right]$ . The level of significance in the data treatment was set at (*ρ* < 0.05). For this purpose, SPSS v.27 software was used.

On the other hand, to perform the qualitative analysis, the technique of polar coordinates was used. This analytical methodology is grounded in the Zsum proposed by Cochran (1954). This principle is based on the notion that the sum of a series of N independent z scores follows a normal distribution characterized by a mean *Z* = 0 and a standard deviation s = √N. Thus, the Zsum statistic is defined as $\mathrm{Zsum}={\Sigma_{i=/}}^{m}\;z/\surd n$ (where n is the number of lags involved). This statistic is crucial, as it facilitates the quantification of the associative strength between different behaviors, as noted by Sackett (1980).

**Figure 1:**
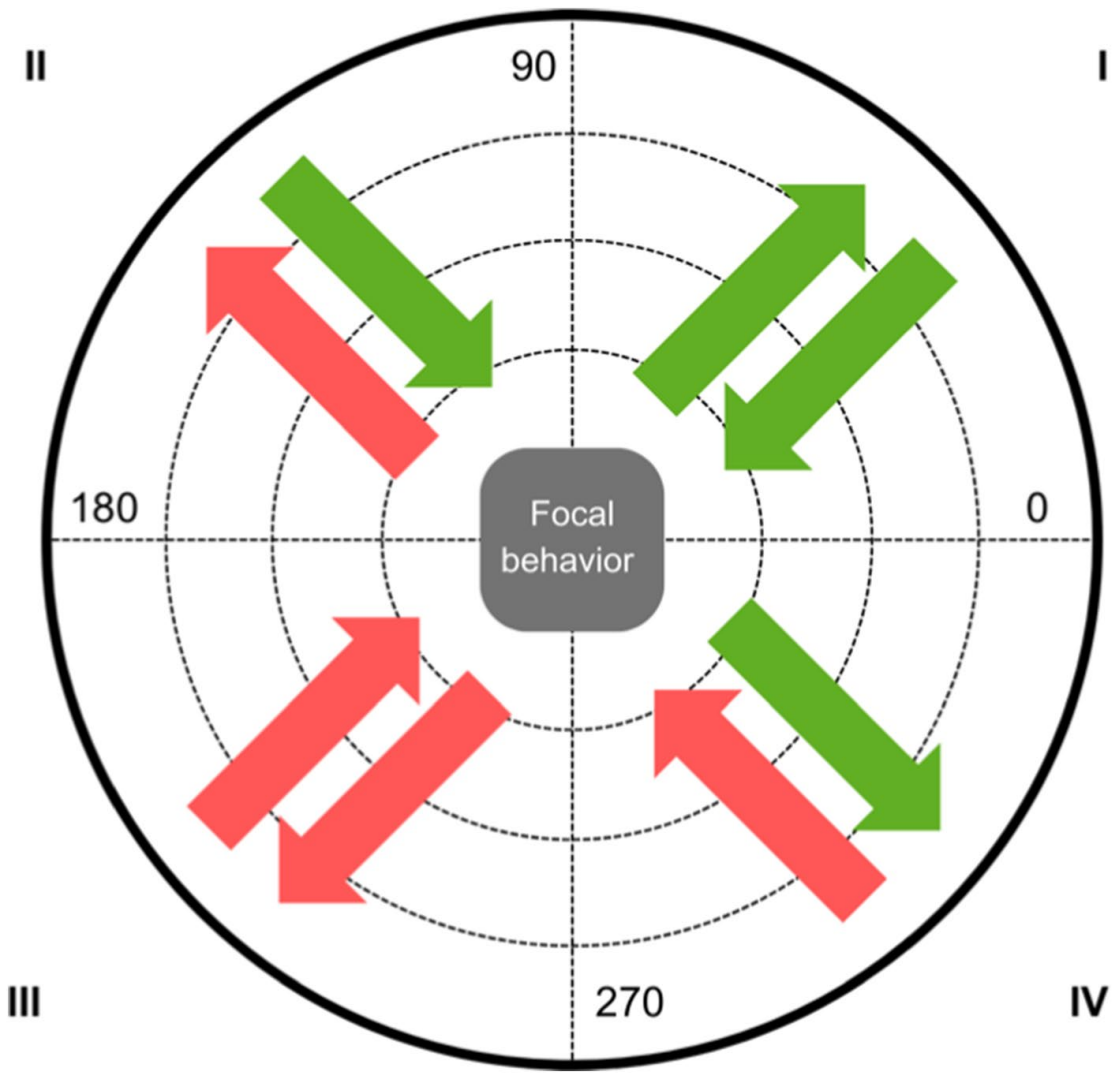
Graphical representation of the excitation or inhibition relationships between the focal behavior and the conditioned behaviors according to the quadrant in which they are located. In green, when it is an excitatory behavior. In red, when it is an inhibitory behavior.

Anguera (1997) subsequently proposed an evolution of this technique by introducing the retrospective perspective into the analysis. Polar coordinate analysis allows the elucidation of the relationships of excitation or inhibition between the focal behavior -that is, the behavior under analysis- and the other behaviors that make up the taxonomic system, referred to as conditioned behaviors. This analysis is carried out both from a prospective approach (from +1 to +5) and from a retrospective perspective (from −1 to −5), resulting in a specific vector for each behavior associated with the focal behavior, each characterized by a particular angle and radius. According to these angular premises, the vector can be located in four sectors or quadrants (Figure 1). If it is in quadrant I, there is mutual excitation between the focal behavior and the conditioned behavior. In quadrant II, the focal behavior inhibits the conditioned behavior, and the conditioned behavior excites the focal behavior. If it is located in quadrant III, there is mutual inhibition of the behaviors. Moreover, if the vector falls into quadrant IV, the focal behavior excites the conditioned behavior, and the conditioned behavior inhibits the focal behavior.

## 3 Results

A total of 21,377 multievents were recorded, understood as each unit of recording performed. This resulted in 2,356 offensive sequences: 630 and 623 from F.C. Barcelona as home and away teams and 526 and 577 from Manchester City, respectively.

Table 3 shows the results of the variables evaluated for each team based on location. The success of actions indicates that there are no significant differences in the Spanish team, with even a slight increase in success when playing away. In contrast, the English team shows a significant decrease in success when playing away matches (*ρ* < 0.001).

**Table 3:** Summary of results based on home and away matches.

Variable		F.C. Barcelona			Manchester City		
		Home	Away	*p*-value	Home	Away	*p*-value
Success of actions	Success	81.6%	81.8%	0.786	82.5%	78.9%	<0.001
	No success	18.4%	18.2%		17.5%	21.1%	
Level of elaboration	Nonexistent	8.9%	9.0%	0.729	7.8%	12.5%	0.004
	Very low	22.5%	26.0%		31.2%	31.0%	
	Low	20.0%	17.3%		14.8%	19.4%	
	Medium	27.6%	26.8%		21.9%	16.6%	
	High	12.7%	11.7%		12.9%	13.3%	
	Very high	4.6%	4.7%		5.9%	3.3%	
	Maximum	3.7%	4.5%		5.5%	3.8%	
Play density	Nonexistent	10.5%	11.2%	0.634	9.7%	14.9%	0.033
	Very low	24.6%	26.0%		33.5%	32.4%	
	Low	20.3%	16.5%		13.9%	16.8%	
	Medium	26.0%	27.0%		20.5%	18.2%	
	High	11.6%	11.3%		12.9%	11.1%	
	Very high	7.0%	8.0%		9.5%	6.6%	
Play duration	Low	21.1%	23.0%	0.665	24.3%	27.2%	0.036
	Medium	22.7%	21.1%		22.6%	27.4%	
	High	56.2%	55.9%		53.0%	45.4%	
Plays with shot/header	Yes	12.7%	9.0%	0.035	13.5%	7.5%	0.001
	No	87.3%	91.0%		86.5%	92.5%	

*ρ < 0.05.

With respect to the level of elaboration, density, and duration, F.C. Barcelona shows similar results regardless of location, with a tendency toward slightly higher values when playing at home. On the other hand, Manchester City presents significant differences in elaboration (*ρ* = 0.004), density (*ρ* = 0.033), and duration of their play (*ρ* = 0.036), with much higher values in home matches than in away matches, where their offensive actions are less elaborate, dense, and enduring.

Finally, concerning the volume of plays with shots or headers on goal, significant differences are observed in both teams. Both F.C. Barcelona (*ρ* = 0.035) and Manchester City (*ρ* = 0.001) have higher levels of action completion when matches are played at home compared to when they play as visitors.

The results obtained through the polar coordinates analysis of the relationships established between the different lines that compose each team’s structure are shown in Table 4 (F.C. Barcelona) and Table 5 (Manchester City). Each line acting as a focal behavior concerning the rest of the team is presented.

**Table 4:** Results of the polar coordinates analysis for different focal categories in relation to the rest of the lines forming FC Barcelona’s structure both home and away.

F.C. Barcelona – Home					F.C. Barcelona – Away				
Focal cat.	Cat.	Quadrant	Radius	Angle	Focal cat.	Cat.	Quadrant	Radius	Angle
GK	DEF	I	5.95**	34	GK	DEF	I	6.4**	43.81
	MID	III	1.97*	235.08		MID	III	4.91**	236.25
	FW	III	4.66**	209.29		FW	III	3.45**	211.26
DEF	GK	I	5.95**	56	DEF	GK	I	6.4**	46.19
	DEF	I	8.71**	45		DEF	I	6.2**	45
	MID	III	3.23**	247.52		MID	III	3.31**	260.51
	FW	III	8.51**	222.78		FW	III	7.22**	211.96
MID	GK	III	1.97*	214.92	MID	GK	III	4.91**	213.75
	DEF	III	3.23**	202.48		DEF	III	3.31**	189.49
	MID	I	2.05*	45		MID	I	1.71**	45
	FW	I	2*	5.12		FW	I	3.92**	8.01
FW	GK	III	4.66**	240.71	FW	GK	III	3.45**	238.74
	DEF	III	8.51**	227.22		DEF	III	7.22**	238.04
	MID	I	2*	84.88		MID	I	3.92**	81.99
	FW	I	9.51**	45		FW	I	6.2**	45

**Table 5:** Results of the polar coordinates analysis for different focal categories in relation to the rest of the lines forming Manchester City’s structure both home and away.

Manchester City – Home					Manchester City – Away				
Focal cat.	Cat.	Quadrant	Radius	Angle	Focal cat.	Cat.	Quadrant	Radius	Angle
GK	DEF	I	6.69**	45.22	GK	DEF	I	4.54**	55.48
	MID	III	3.62**	219.39		MID	IV	3.14**	290.42
	FW	III	5.18**	232.47		FW	III	6.48**	214.14
DEF	GK	I	6.69**	44.78	DEF	GK	I	4.54**	34.52
	DEF	I	10.18**	45		DEF	I	10.09**	45
	MID	III	4.84**	208.25		MID	III	4.27**	215.19
	FW	III	10.4**	235.73		FW	III	9.55**	229.16
MID	GK	III	3.62**	230.61	MID	GK	II	3.14**	159.58
	DEF	III	4.84**	241.75		DEF	III	4.27**	234.81
	MID	I	3.64**	45		MID	I	3.93**	45
	FW	I	3.89**	75.69		FW	I	1.66	28.15
FW	GK	III	5.18**	217.53	FW	GK	III	6.48**	235.86
	DEF	III	10.4**	214.27		DEF	III	9.55**	220.84
	MID	I	3.89**	14.31		MID	I	1.66	61.85
	FW	I	10.81**	45		FW	I	12.33**	45

First, the ‘GK’ (Goalkeeper) category as the focal behavior in both teams, in contrast to the ‘DEF’ (Defenders), ‘MID’ (Midfielders), and ‘FW’ (Forwards) categories identified as conditioned behaviors. This approach was designed to examine the goalkeeper’s predisposition to play in relation to the other lines that compose the game structure during matches.

**Figure 2:**
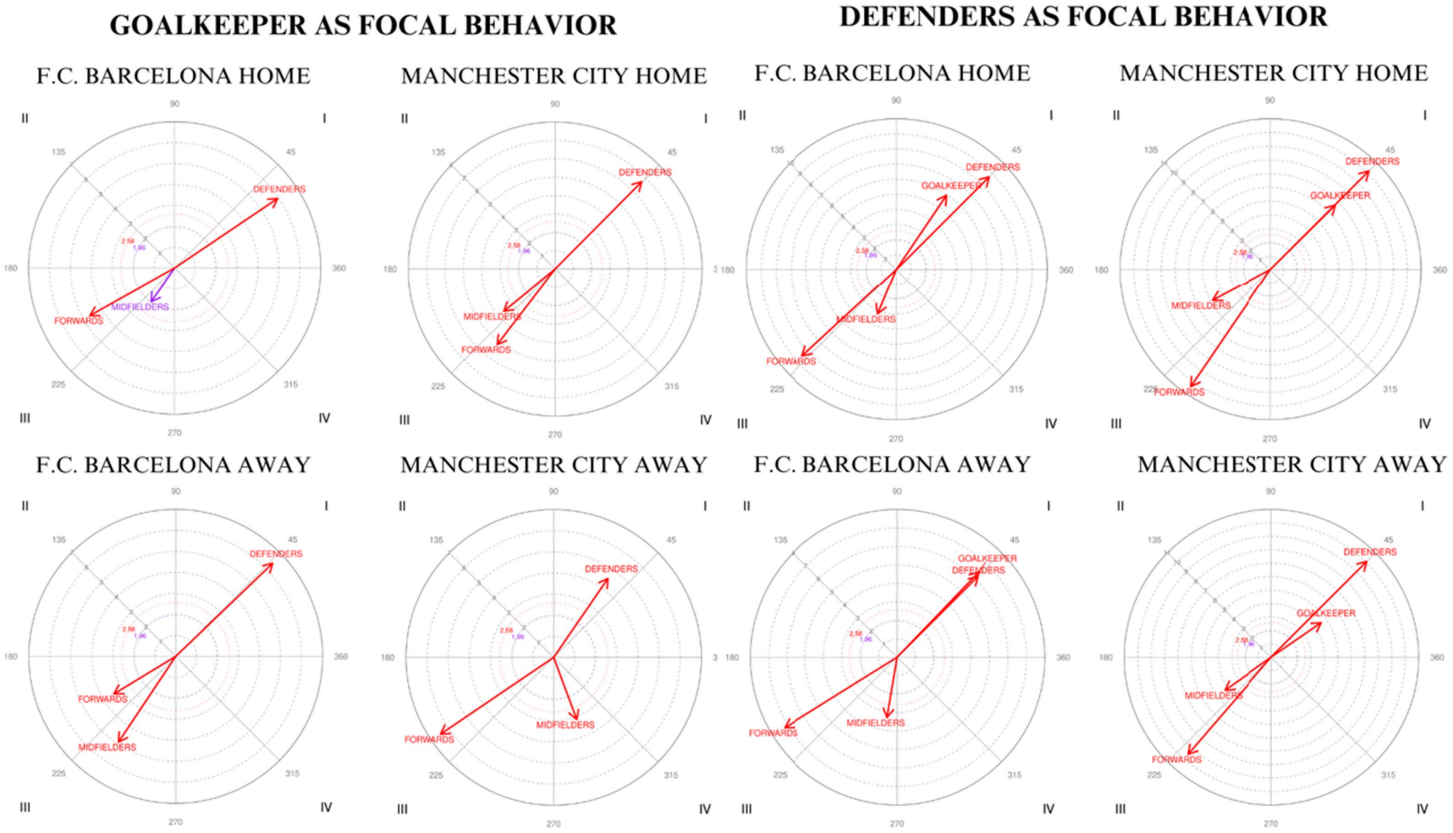
Behavioral map representation establishing the goalkeeper and defense categories as focal behaviors in relation to the lines structuring the team.

With respect to the results (Table 4 and Figure 2), in the case of F.C. Barcelona, the conditioned category ‘DEF’ is located in quadrant I, with a radius length of 5.95 and angle of 34° when playing at home and a radius of 6.4 and angle of 43.81° when playing away. This finding indicates that focal behavior activates the presence of conditioned behavior in both the prospective and retrospective planes. The ‘MID’ and ‘FW’ categories are located in quadrant III in both locations, indicating that the focal behavior inhibits the presence of the conditioned behavior in both planes. The values associated with these categories are radii of 1.97 and 4.66 and angles of 235.08° and 209.29°, respectively, when playing at home and radii of 4.91 and 3.45, with angles of 236.25° and 211.26°, respectively, when playing away.

In the case of Manchester City (Table 5 and Figure 2), when the match is at home, the ‘DEF’ category is located in quadrant I, with a radius of 6.69 and an angle of 45.22°, and the ‘MID’ and ‘FW’ categories are positioned in quadrant III, with radii of 3.62 and 5.18 and angles of 219.39° and 232.47°, respectively. Conversely, when playing away, the ‘DEF’ and ‘FW’ categories remain in the same quadrants (radii of 4.54 and 6.48 and angles of 55.48° and 214.14°), but the ‘MID’ category is in quadrant IV with a radius of 3.14 and an angle of 290.42°. This implies that focal behavior activates the presence of conditioned behavior in the prospective plane but not in the retrospective plane.

Second, the focal behavior was set as ‘DEF’ (Defenders) and related to the remaining categories that configure the team’s structure, including the defensive line itself: ‘GK’, ‘DEF’, ‘MID’, and ‘FW’. The objective of this approach is to investigate the playing predisposition exhibited by the defensive line in relation to the other lines that make up the team’s tactical structure during matches.

The results obtained (Table 4 and Figure 2) for F.C. Barcelona show that the categories ‘GK’ and ‘DEF’ are located in quadrant I for both home and away matches, indicating that focal behavior activates the presence of conditioned behavior in both the prospective and retrospective planes. The corresponding values are radii of 5.95 and 8.71 with angles of 56° and 45°, respectively, for home matches and radii of 6.4 and 6.2 with angles of 46.10° and 45° when playing away. In contrast, the categories ‘MID’ and ‘FW’ are situated in both cases in quadrant III, where the focal behavior inhibits the presence of the conditioned behavior in both planes. The values associated with these categories are radii of 3.23 for midfielders and 8.51 for forwards, with angles of 247.52° and 222.78° at home, and radii of 3.31 and 7.22, with angles of 260.51° and 211.96° away, respectively.

In the case of Manchester City (Table 5 and Figure 2), the categories ‘GK’ and ‘DEF’ are located in quadrant I, with radii of 6.69 and 10.18 and angles of 44.78° and 45° at home and radii of 4.54 and 10.09 with angles of 34.52° and 45° when playing away. The categories ‘MID’ and ‘FW’, on the other hand, are found in quadrant III, with radii of 4.84 and 10.4 and angles of 208.25° and 235.73° at home and radii of 4.27 and 9.55 with angles of 215.19° and 229.16° when playing away.

In the third segment of the analysis, the midfielders (MID) were established as the focal behavior, both in relation to themselves and to the other lines on the field: ‘GK’, ‘DEF’, ‘MID’, and ‘FW’. This approach aims to evaluate how the midfield line interacts with the other positions that compose the tactical structure throughout the match.

**Figure 3:**
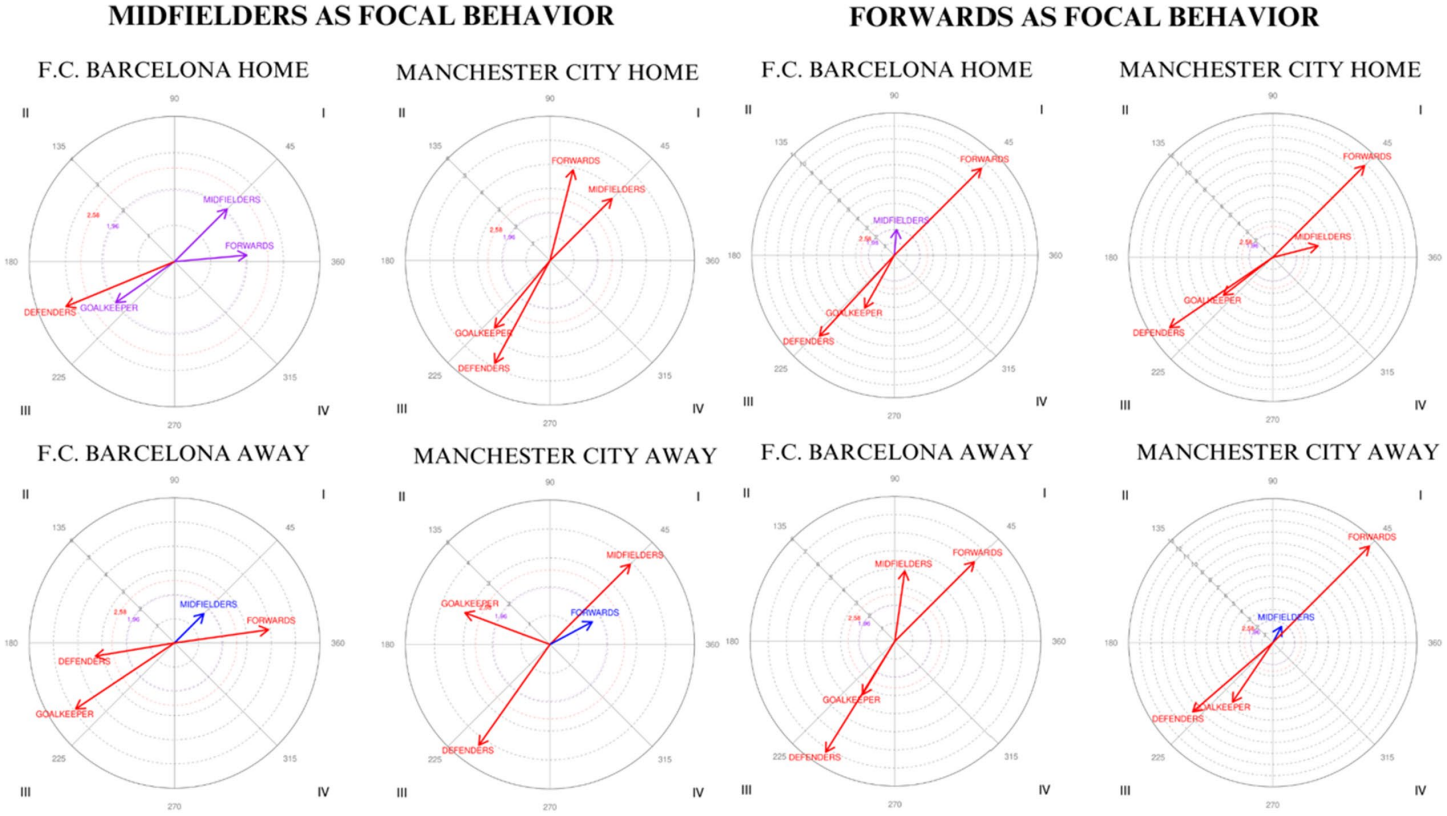
Behavioral map representation establishing the midfielder and forward categories as focal behaviors in relation to the lines structuring the team.

The results obtained (Table 4 and Figure 3) for F.C. Barcelona indicate that the categories ‘GK’ and ‘DEF’ are located in quadrant III, both at home and away, with radii of 1.97 and 4.91 and angles of 214.92° and 213.75°, respectively, for the ‘GK’ category, and radii of 3.23 and 3.31, with angles of 202.48° and 189.49°, respectively, for the ‘DEF’ category. In this context, focal behavior inhibits the presence of conditioned behavior in both the prospective and retrospective planes. On the other hand, the categories ‘MID’ and ‘FW’ are in quadrant I, activating the conditioned behavior in both planes. The corresponding values are a radius of 2.05 and an angle of 45° for the midfielders, a radius of 2 and an angle of 5.12° for the forwards in home matches, and radii of 1.71 and 3.92 with angles of 45° and 8.01° for the ‘MID’ and ‘FW’ when playing away.

With respect to Manchester City (Table 5 and Figure 3), the categories ‘GK’ and ‘DEF’ are situated in quadrant III, with radii of 3.62 and 4.84 and angles of 230.61° and 241.75°, respectively, when playing at home and radii of 3.14 and 4.27, with angles of 159.58° and 234.81°, respectively, when playing away. The categories ‘MID’ and ‘FW’, in contrast, appear in quadrant I in both locations, with a radius of 3.64 and an angle of 45° for the former, a radius of 3.89 and an angle of 75.69° for the latter at home, and radii of 3.93 and 1.66 with angles of 45° and 28.15°, respectively, when playing away.

In the fourth and final segment of the study, the forwards (FW) category was adopted as the focal behavior to analyze its interaction with the other team lines along with itself: ‘GK’, ‘DEF’, ‘MID’, and ‘FW’. This approach aims to explore the tactical predisposition of the forward line in relation to the other positions that compose the team’s structure throughout the match.

In the case of F.C. Barcelona, as represented in Table 4 and Figure 3, the categories ‘GK’ and ‘DEF’ are located in quadrant III both at home and away. This finding indicates that focal behavior, represented by the forwards, inhibits the presence of conditioned behavior in both the prospective and retrospective planes. The specific values reflect radius lengths of 4.66 and 8.51 and angles of 240.71° and 227.22°, respectively, when playing at home and 3.45 and 7.22 with angles of 238.74° and 238.04°, respectively, when playing away. On the other hand, the categories ‘MID’ and ‘FW’ are found in quadrant I, which means that the focal behavior activates the presence of the conditioned behavior in both planes. With respect to the midfield line, values of 2 in length and an angle of 84.88° are observed, whereas for the forwards, the values are 9.51 in length and 45° in angle at home. Conversely, when playing as visitors, the radii are 3.92 and 6.2, with angles of 81.99° and 45°, respectively, for these two conditioned categories.

Regarding Manchester City, the categories ‘GK’ and ‘DEF’ are also positioned in quadrant III, with radius lengths of 5.18 and 10.4 and angles of 217.53° and 214.27° when playing at home and radii of 6.48 and 9.55 with angles of 235.86° and 220.84°, respectively, when playing away. Moreover, the categories ‘MID’ and ‘FW’ are located in quadrant I, with radius lengths of 3.89 and 10.81, angles of 14.31° and 45° at home, and lengths of 1.66 and 12.33 along with angles of 61.85° and 45° when playing away from their own stadium.

## 4 Discussion

The objective of the present research is to determine whether the playing style developed by teams coached by Pep Guardiola remains stable regardless of the match location or if it is influenced by contextual variables that alter its game dynamics. The study reveals notable differences in the playing methodology of F.C. Barcelona and Manchester City under Guardiola’s leadership: the Spanish team’s playing style appears to be stable despite changes in setting, whereas the English team’s tactics show significant adaptability depending on whether they play at home or away.

With respect to the descriptive results, Manchester City’s evaluated parameters exhibit significant variations on the basis of match location, in stark contrast to F.C. Barcelona, whose comparative analysis shows remarkable consistency, except in the aspect of shots on goal, where a significant difference is identified. The variability in the playing styles between both teams can be attributed to multiple factors.

Initially, it was worth highlighting the divergence in the rosters of both clubs. F.C. Barcelona, during a period when its playing style and dominance were the subject of extensive academic analysis (Buldú et al., 2019; Chassy, 2013), had players ranked among the best in European football, whose skills have been the subject of various investigations (Castañer et al., 2016; Lapresa et al., 2020; Maneiro et al., 2019). These teams have already been studied in previous research (Pueyo et al., 2024), confirming the difference in playing style between the two teams. On the other hand, considering the competitive context of each team is essential. Spain’s Primera División is distinguished by more combinative play and prolonged ball possession (González-Rodenas et al., 2023), while the English Premier League leans toward a more direct and vertical style, influenced by the intense pressing that characterizes teams in this league (Mitrotasios et al., 2019). The differences between these competitions have been extensively examined in the literature (Cooper and Pulling, 2020; Nagy et al., 2023), providing a basis for understanding the discrepancies in the methods used by teams participating in both competitions.

Moreover, the idea that playing at home confers an advantage in football is well accepted in the literature, with evident differences in the number of goals scored, the effectiveness of technical actions, and the probability of victory at the end of the match (Almeida et al., 2014; Diana et al., 2017; Sarmento et al., 2014). In the case of F.C. Barcelona, indicators of success in actions performed, the elaboration of plays, as well as their density and duration, maintain notable consistency both in matches at their stadium and those played away. In contrast, Manchester City’s analysis reveals a significant increase in the effectiveness, elaboration, density, and durability of plays when they compete on their own ground. This pattern suggests that playing at home enhances the team’s ability to generate more complex and sustained sequences of play that culminate more successfully. Additionally, the last parameter examined, referring to the percentage of offensive flow generated, corroborates that both teams, when playing at home, increase their ability to finish play, which is essential for achieving the ultimate goal in football: scoring.

The exploration of interline relationships through polar coordinate analysis -a technique previously applied in this context (Maneiro et al., 2018) corroborates the observed trends. A notable consistency is highlighted in the defensive line of both teams, which, despite experiencing variations in intensity, maintains a uniform vectorial distribution, suggesting that the function of these players with the ball remains unaltered, regardless of match location. However, a more detailed examination of the other lines constituting the teams reveals significant contrasts, especially in Manchester City. This analysis reveals that, in away game situations, the team’s goalkeeper establishes connections with midfielders predominantly in quadrant IV, unlike quadrant III, which is observed when playing at home, where their relationships are mutually inhibited. This condition may indicate that, under high-pressure situations, the goalkeeper plays with the midfield line that drops back to receive the ball or opts to bypass the defensive line, facilitating forward ball movement through direct links with midfielders as a strategy to overcome opposing pressure lines.

Focusing on the midfield line, similar vectorial trends are observed in F.C. Barcelona, whereas notable differences are detected in Manchester City. The midfielders of the Catalan team maintain interactions mainly with their own midfield line and with the forwards, showing a greater preference for the latter in away matches, suggesting a tendency toward greater verticality in play. In contrast, the English team, although exhibiting behavior similar to that of F.C. Barcelona in home matches, shows a different relationship when playing away, with the goalkeeper positioning in quadrant II, reflecting the reception of the ball directly from the goalkeeper as a tactical resource. Although there is a tendency toward interaction with the forward line in quadrant I, it does not reach statistical significance, indicating that, while present, the relationship between these two lines is not predominantly strong.

Regarding the forward line as the focal behavior, the vectors consistently occupy the same quadrants under all circumstances, differing only in the intensity of their interactions. The relationship between forwards and midfielders, situated in quadrant I, reflects mutual excitation with the focal behavior. However, the intensity of this interaction with F.C. Barcelona’s midfielders in home matches, although significant, is less pronounced than that in away matches. In contrast, Manchester City demonstrates significant connections both with forwards and midfielders in quadrant I and with defenders and the goalkeeper in quadrant III in home matches. However, in away game contexts, the significant relationship with the midfielders diminishes, with the interaction among forwards predominating when they are in possession of the ball.

The study is not without limitations in adequately contextualizing the findings. One relates to the sample size employed. The analysis focused on ten matches of each team, played against the top teams in their respective competitions, suggesting that the results obtained should be interpreted with caution and considered preliminary until they can be complemented and validated by future studies with larger samples against rivals of various standings and in different competitions, thereby expanding the scope of analysis and corroborating the observed findings. Additionally, this study did not explicitly examine the impact that the opponent team’s behavior, particularly their ball pressure at each moment, may have on the playing style. Lastly, this manuscript lacks other analyses that could have been of interest, such as T-Patterns (Pic and Jonsson, 2021), which might have yielded clearer results regarding the objective of the study. Future research may focus on exploring how the location and characteristics of different competitions affect teams’ tactical approaches, as well as comparing the strategies adopted by Pep Guardiola with those of other coaches of similar styles to assess adaptability in different contexts.

## 5 Conclusion

The aim of this research was to analyze the variations in the playing styles of two teams coached by Pep Guardiola to determine differences in their performance on the basis of whether they played their matches at home or away. The results indicate that F.C. Barcelona’s playing methodology remains consistent, highlighting greater completion of plays in their own stadium and notable stability in the interrelations among the various positional lines that compose their formation, regardless of match location. In contrast, Manchester City shows significant variability in all evaluated aspects, offering a higher percentage of success in their actions, as well as more elaborate, dense, and prolonged offensive sequences when playing at home. Furthermore, with the exception of the defensive line, the other positional lines of the team show variations in their interrelations depending on the match location. Therefore, the coach under study does not always maintain a singular playing style, and, depending on different competitive variables—such as the type of competition, the squad, or match location—it becomes necessary to adapt the playing style to the demands of the situation.

The practical applications arising from this study involve equipping teams with tactical alternatives on the basis of the match context. Playing at home or away can constitute a contextual factor that affects the development and style of play; therefore, preparing players to confront these performance constraints will be important for increasing the chances of victory.

## Data Availability

The raw data supporting the conclusions of this article will be made available by the authors, without undue reservation.
